# Analysis of Incentive Policies and Initiatives on Orphan Drug Development in China: Challenges, Reforms and Implications

**DOI:** 10.1186/s13023-023-02684-8

**Published:** 2023-07-27

**Authors:** Zhiyao Zhao, Zhongyang Pei, Anxia Hu, Yuhui Zhang, Jing Chen

**Affiliations:** 1 School of Foreign Languages, China Pharmaceutical University, Nanjing, China; 2 Institute of Epidemiology and Health Care, University College London, London, UK; 3 School of Management, Shanxi Medical University, Taiyuan, China; 4 School of Health Services and Management, Shanxi University of Chinese Medicine, Taiyuan, China

**Keywords:** Orphan drug development, Rare disease, Incentive policy, Reform, Implication

## Abstract

**Objectives:**

Rare diseases are a global public health issue with a more pressing situation in China. Unfortunately, the relevant research and development in this country are still in its infancy, leading to limited drug accessibility. In view of this, the Chinese government has taken a series of countermeasures to promote orphan drug R&D in recent years, which has presented encouraging results. This paper aims to review incentive policies and funding initiatives formulated by the Chinese government and examine their implications on orphan drug R&D.

**Methods:**

Policies targeting orphan drug R&D during 2012–2022 were retrieved from the relevant official websites, categorized into different themes and analyzed for the contents. Data on government funding, drug approval, clinical trial approval and orphan drug designation were collected through internet search to analyze the implications of those incentive policies and initiatives on orphan drug R&D in China.

**Results:**

A total of 20 relevant policy documents were identified and five major themes were revealed through content analysis, including national strategy, expedited approval, safety and efficacy requirements, data protection and technical support. The government input in orphan drug R&D has witnessed a steady annual increase. Driven by those incentives, the numbers of orphan drugs approved for marketing and drug candidates entering clinical studies are increasing year by year, and more domestic pharmaceutical companies are actively involved in the R&D of orphan drugs.

**Conclusions:**

Orphan drug development in China is growing rapidly under the stimulation of incentive regulatory policies and more investment in researches. China is working toward a more standardized and comprehensive rare disease ecosystem. However, there are still some challenges, such as the lack of sufficient financial support and the call for systematic legislation on rare diseases, to be addressed for future success.

## Introduction

The exact definition of rare disease varies across different countries, depending on the population size, disease epidemiology and available treatment options. At present, more than 7,000 rare diseases have been identified worldwide [1], accounting for approximately 10% of total disease prevalence [2], and the number is still increasing. However, a large number of patients often remain undiagnosed or even misdiagnosed [3], and fewer than 10% receive disease-specific treatment [4]. The number of rare disease patients in China is estimated to be more than 20 million, with more than 200,000 new cases each year [5]. Despite the low prevalence and incidence of each specific disease, the unmet medical need of rare disease patients in China is huge due to the large population base, making it a major public health issue that cannot be ignored.

Compared with more widespread chronic diseases, the markets of orphan drugs are so small that pursuing these therapies is viewed as unprofitable by pharmaceutical companies and these medical products are consequently “orphaned” [6]. Even in western countries, orphan drug R&D had long been ignored by the pharmaceutical industry before the establishment of a range of governmental policies and initiatives that include financial subsidies in support of R&D activities by pharmaceutical companies, tax credits, fee waivers, expedited approval, and market exclusivity. These efforts, together with the interest and commitment of a wider range of stakeholders including researchers, health care professionals and patient advocacy organizations, have led to significant progress in the R&D and marketing of innovative orphan drugs. Since 2000, 2,552 orphan medicinal product designations have been issued by the European Commission, of which 207 have been authorized for marketing so far [7]. In the US, since the enactment of the Orphan Drug Act in 1983, over 5000 medical products have received orphan drug designation [8], and at the end of 2020, 559 drugs had been approved for 943 orphan indications [9]. In 2020, the global market size of orphan drugs had reached $122,720 million and was projected to reach $241,610 million by 2027[10]. The steady growth in the number of orphan drug approvals and market share worldwide indicates a new era of rare disease research and orphan drug innovation.

“Insufficient drug accessibility” is the most pressing issue for rare disease patients in China [11–13]. Although orphan drug R&D in China still lags behind developed countries, some encouraging progresses have been made recently due to multiple contributors, including the growth of domestic pharmaceutical industry (currently ranking second in the world market), important efforts and support by the Chinese government and initiatives from a wider range of stakeholders (development of relevant consortia and patient advocacy organizations). Among them, supportive policies and initiatives from the government, ranging from national strategy and preferential regulatory policies to data protection and protocol assistance and further to increased funding, have played the most direct and important role. China is determined to play more important roles in the global fight against rare diseases, turning the challenges into opportunities for both domestic and international companies. The aim of this study is to systematically review incentive policies and funding initiatives targeting orphan drug R&D in China during 2012–2022 and analyze current development status, major achievements, and existing challenges. We hope to provide policy makers with a critical understanding of the historic evolution of orphan drug policy, the unique situation of rare diseases in China and the impacts of policy reforms implemented so far for further policy refinement, offer drug regulators some insights into closer collaboration with the industry and the establishment of more cost-effective regulatory framework to enhance orphan drug R&D and encourage drug developers both at home and abroad to make more contributions to address this unmet medical need.

## Methods

### Data retrieval

For policy analysis, relevant government documents were researched, reviewed and retrieved on the official website of the National Medical Products Administration (NMPA) (https://www.nmpa.gov.cn/index.html), the Center for Drug Evaluation (CDE) (https://www.cde.org.cn/), the State Council (https://www.gov.cn/) and the State Administration for Market Regulation (SAMR) (https://www.samr.gov.cn/) using keywords of “rare disease” and “orphan drug” within the time range of 2012–2022. All documents that are described as guidelines, announcements, opinions, regulations, provisions and policies and related to the various aspects of orphan drug R&D were screened and selected for further identification of policy themes and content analysis. All documents were screened, selected, reviewed and cross-checked by independent researchers manually. All discrepancies in selection were settled during consensus meetings with the participation of most of the authors.

For funding information, the websites of “inquiry of projects funded by the National Natural Science Foundation of China (NSFC)” (http://fund.zsci.com.cn/Index/index.html) and LetPub (https://www.letpub.com.cn/index.php?page=grant) were searched based on the National List of Rare Diseases (NLRD) issued by the National Health Commission in May 2018 for the period from January 1, 2012 to December 31, 2021. For the information of drug approval and orphan drug designation, the official websites of NMPA and the U.S. Food and Drug Administration (FDA) were searched, including their online databases, news reports, annual reports and bulletins. Clinical trial data of orphan drugs were extracted from the Registration and Information Disclosure Platform for Drug Clinical Studies (http://www.chinadrugtrials.org.cn) of NMPA. Only drugs that are recognized as on-label use for specific rare disease indications have been included in the analyses.

### Content analysis and data analysis

Themes of orphan drug policies were identified and categorized based on relevant texts extracted from selected documents. Cross-checking was conducted by two researchers independently, and all discrepancies in theme identification and categorization were settled during consensus meetings. A time series analysis was conducted to assess the trends in funding initiatives, drug approval, clinical trial approval and orphan drug designation. Qualitative data analysis was performed using SPSS software ver. 22.0 (IBM, Armonk, NY, USA) and basic descriptive statistics was performed for funding characteristics, including total counts and percentages.

## Results

### Identified themes and characteristics of incentive policies on Orphan Drug Development

A total of 20 relevant policy documents were retrieved and selected from the official website of NMPA in the period of 2012–2022, which are reviewed and analyzed in Table 1 in a chronological manner. Apart from laws and regulations, these R&D incentives are more frequently described in various announcements, guidelines, notices and opinions, which prioritize orphan drug development and provide details for implementation. These incentives mainly focus on 5 themes (further divided into 9 subthemes), including national strategy, expedited approval (accelerated review and approval, priority review and approval, conditional approval), safety and efficacy requirements (clinical trial exemption, acceptance of overseas clinical trial data), exclusivity (data protection, market exclusivity) and technical support (protocol assistance, scientific advice/consultation).

**Table 1:** Incentive policies on orphan drug development

Effective time	Title	Theme	Subtheme	Related Content
2012-1	The 12th Five-Year Plan for National Drug Safety [14]	National strategy	National strategy	Strengthen the quality supervision of a drug product throughout its lifecycle, and encourage the R&D of orphan drugs and pediatric dosage forms.
2013-2	Opinions on Deepening the Reform of the Review & Approval System and Encouraging Drug Innovation [15]	Expedited approval	Priority review	Implement priority review for orphan drugs; accelerate the registration application of innovative drugs for rare diseases.
2015-8	Opinions of the State Council on the Reform of Review and Approval System of Drugs and Medical Devices [16]	Expedited approval	Accelerated review and approval	Accelerate the review and approval of innovative drugs for AIDS, malignant tumors, major infectious diseases and rare diseases.
2015-11	Announcement on Certain Relevant Policies on the Review and Approval of Drug Registration [17]	Expedited approval	Accelerated review and approval	Set up a separate queue to accelerate the review and approval of innovative drugs for the prevention or treatment of HIV/AIDS, malignant tumors, severe infectious diseases, and rare diseases.
2016-2	Opinions on Solving the Backlog of Drug Registration Applications and Implementing Priority Review and Approval [18]	Expedited approval; technical support;; safety and efficacy requirements	Priority review and approval; protocol assistance; scientific advice/consultation; clinical trial exemption	Drugs for the prevention and treatment of rare diseases can be granted priority review and approval; the CDE provides scientific advice for the development of clinical protocol and consultations for decision making. For orphan drugs, applicants can apply for abbreviation or waiver of clinical trials, and the CDE will give review advice based on technical review needs and the actual situation of Chinese patients.
2016-4	Notice of the CPC Central Committee and the State Council on Issuing the Key Tasks of Deepening the Health Care System Reform [19]	Expedited approval	Accelerated review and approval	Further unblock the green channels for the review and approval of drugs for rare diseases or in urgent clinical need or for the special patient population, and accelerate the progress of registration review.
2017-2	13th Five-Year Plan for National Drug Safety [20]	Expedited approval	Accelerated review and approval	Accelerate review and approval of drugs and medical devices for rare diseases and speed up the launch of orphan drugs with independent intellectual property rights.
2017-10	Opinions on Deepening the Reform on Review and Approval System and Encouraging Innovation on Drugs and Medical Devices [21]	Safety and efficacy requirements, exclusivity, expedited approval	Clinical trial exemption; data protection; conditional approval	Propose to publish a national list of rare diseases and establish a registration system for patients with rare diseases; applicants for drugs and medical devices for rare diseases can apply for waiver or abbreviation of clinical trials and submit an application for the protection of independent and yet undisclosed study data; marketing application of the same medical product developed by other applicants will not be approved during the data protection period; conditional approval can be granted for drugs and medical devices for rare diseases approved overseas.
2017-12	Opinions of the China Food and Drug Administration on Priority Review and Approval to Encourage Drug Innovation [22]	Expedited approval; safety and efficacy requirements;	Priority review and approval; clinical trial exemption;	Priority review and approval can be granted for orphan drugs with significant clinical advantages. Applicants can apply for abbreviation or waiver of clinical trials for orphan drugs.
2018-4	Interim Measures for Implementing Pharmaceutical Study Data Protection (Draft for Public Review) [23]	Exclusivity	Data protection	Orphan drugs are included in the scope of data protection and can be entitled to a six-year data exclusivity upon the date of first approval of the relevant indication in China.
2018-5	Announcement on Optimizing Review & Approval of Drug Registration[24]	Safety and efficacy requirements	Acceptance of overseas clinical trial data	Applicants of orphan drugs approved overseas can directly submit registration application by using overseas clinical trial data if the ethnic/racial difference has been proved to be non-existent.
2018-7	Technical Guidelines for Accepting Overseas Clinical Trial Data of Drugs [25]	Safety and efficacy requirements	Acceptance of overseas clinical trial data	Based on the quality of overseas clinical trial data, its acceptance by NMPA will be divided into fully accepted, partially accepted and rejected. For application of orphan drugs whose overseas clinical trial data are categorized as “partially accepted”, the NMPA may grant a conditional acceptance of the overseas clinical trial data subject to the collection of further efficacy and safety data for evaluation after market authorization.
2018-10	The Review and Approval Process for Overseas Approved New Drugs with Urgent Clinical Need [26]	Expedited approval	Accelerated review and approval	Establish special channels for the review and approval of orphan drugs that have been marketed in the US, EU or Japan in the past decade but not yet in China; technical review of orphan drugs should be completed within three months (six-month for other overseas approved new drugs) upon the reception of application dossier.
2019-5	Key Considerations in Using Real-World Evidence to Support Drug Development (Draft for Public Review) [27]	Technical support	Protocol assistance	Encourage the use of real-world evidence to evaluate drug safety and efficacy; for clinical trials of orphan drugs, external controls based on real-world data in natural disease cohorts can be considered.
2019-12	Drug Administration Act [28]	National strategy	National strategy	Encourage the development of new drugs indicated for rare diseases.
2020-1	Provisions for Drug Registration [29]	Expedited approval	Priority review and approval	Applicants of innovative drugs and modified new drugs indicated for rare diseases can apply for priority review and approval for marketing authorization; for urgently needed orphan drugs that have been marketed overseas but not yet in China, the review procedure should be completed within 70 days.
2020-12	Administrative Measures for Communication of Drug R&D and Technical Review [30]	Technical support	Scientific advice/consultation	Provide applicants with a variety of proactive communication channels such as face-to-face meetings, video conferences and written responses for consultation regarding key technical issues that are not covered by currently issued guidelines for drug R&D and evaluation; applicants of orphan drugs can propose a communication meeting with CDE to jointly discuss relevant technical issues, particularly regarding priority review and approval. The consensus reached by both parties can be used as an important reference for drug development and evaluation.
2021-12	Technical Guidelines for Clinical Development of Orphan Drugs [31]	Technical support	Scientific advice/consultation	The CDE suggests innovative and widely accepted approaches to overcome common challenges in clinical development of orphan drugs to further improve the efficiency in orphan drug development.
2022-5	Implementing Regulations of the Drug Administration Law of the People’s Republic of China (Draft for Public Review) [32]	Expedited approval ; exclusivity	Priority review and approval; market exclusivity	Encourage the R&D of orphan drugs and indication expansion of existing drugs to cover rare diseases; implement priority review and approval for orphan drugs with urgent clinical needs and speed up the marketing process; grant 7-year exclusivity for approved orphan drugs to prohibit generic manufacturers from securing a marketing authorization.
2022-6	Guidelines for Statistics of Clinical Research of Orphan Drugs (Draft for Public Review) [33]	Technical support	Scientific advice/consultation	The CDE provides guidance for sponsors to conduct rare disease clinical research, especially by addressing key statistical issues in research.
CDE, Center for Drug Evaluation; NMPA, National Medical Products Administration				

Among those themes, “expedited approval” is the most identified one. Starting from 2013, several policies have been issued to accelerate the review and approval process for orphan drugs. The *Review and Approval Process for Overseas Approved New Drugs with Urgent Clinical Need* issued in October 2018 illustrates how the expedited approval procedures work for urgently needed drugs approved overseas, including orphan drugs [26]. The technical review timeline for orphan drugs that have been marketed in the US, EU or Japan in the past decade but not yet in China can be shortened to 3 months, while the standard technical review time for a new drug is 120–160 days. One month later, the NMPA issued its first batch of 48 drugs that have been approved overseas and urgently needed in China, 20 of which are indicated for rare diseases. The second and the third batch were released in 2019 and 2022, containing 17 (out of 26) and 3 (out of 7) orphan drugs, respectively. Up to now, 46 drugs in the three batches have been approved in China and 21 are orphan drugs. In the *Provisions for Drug Registration* promulgated by SAMR on January 22, 2020, four expedited approval pathways were officially established in China, namely, breakthrough therapy, conditional approval, priority review and approval and special approval [29]. Although all of the first three pathways are applicable to orphan drugs meeting specified criteria, priority review and approval is the most exercised one. Sponsors of innovative drugs and modified new drugs indicated for rare diseases can apply for priority review and approval for marketing authorization. With this designation, the time limit for review and approval of marketing authorization is 130 days, which is further shortened to 70 days for urgently needed orphan drugs that have been marketed overseas but not yet in China.

The second most identified theme is “safety and efficacy requirements”. Compared with the development of non-orphan drugs, the development of drugs targeting rare diseases is always hindered by the limited number of patients and insufficient knowledge of disease pathophysiology. Consequently, measures should be taken to have more efficient assessments of the safety and efficacy of orphan drugs based on limited clinical trial data. In several Opinions that have been issued by NMPA since February, 2016, sponsors of orphan drugs are allowed to apply for abbreviation or waiver of clinical trials [18, 22, 24, 25]. For orphan drugs approved overseas, their registration application can be based on overseas clinical trial data if the ethnic/racial difference has been proved to be non-existent. Based on the quality of overseas clinical trial data, its acceptance by NMPA will be divided into “fully accepted”, “partially accepted” and “rejected”. For application with “partially accepted” clinical data, the NMPA may grant a conditional acceptance of the overseas clinical trial data subject to the collection of further efficacy and safety data for evaluation after market authorization. In 2017, the world’s first orphan drug for the treatment of Pompe disease, Myozyme, was approved for marketing in China under conditional approval with the exemption of phase III clinical trial. The conditional approval was based on several finished phase II clinical trials and five ongoing clinical trials, as well as the experience of clinical application in foreign countries. The applicant was required to conduct a post-marketing evaluation of drug safety and efficacy in no less than 50 Chinese patients for at least one year.

Data protection has also become part of the toolbox used by the Chinese government to promote orphan drug R&D. The *Interim Measures for Implementing Pharmaceutical Study Data Protection* grants a six-year data protection period for orphan drugs upon the date of first approval of the relevant indication in China [23]. Marketing application of the same medical product developed by other applicants will not be approved during this period, which is conducive to reducing the competition from generic drugs. The newly issued *Implementing Regulations of the Drug Administration Law of the People’s Republic of China* in May 2022 proposes significant changes to the current 2019 version [32]. One of the highlights is to entitle innovative orphan drugs to a market exclusivity period of up to seven years, conditional upon the market authorization holder’s undertaking to guarantee the supply of the drug.

In recent years, the NMPA has actively explored more basic technical support for orphan drug development. In December 2021, China’s CDE released its first technical guidelines for the clinical development of orphan drugs, demonstrating a concrete step toward tackling rare diseases [31]. The CDE suggests innovative and widely accepted approaches to overcome common challenges in the clinical development of orphan drugs, including clinical trial design, data collection, indication expansion etc. Since the development of orphan drugs is still in its infancy in China, these guidelines are urgently needed by domestic pharmaceutical companies for more efficient drug innovation. In addition, the NMPA tries to provide more convenient and faster channels of communication between applicants and administrative authorities during drug registration and review to assist the development of clinical protocol and decision-making on key technical issues.

### Initiatives to Enhance R&D Input for Orphan Drugs

**Fig. 1:**
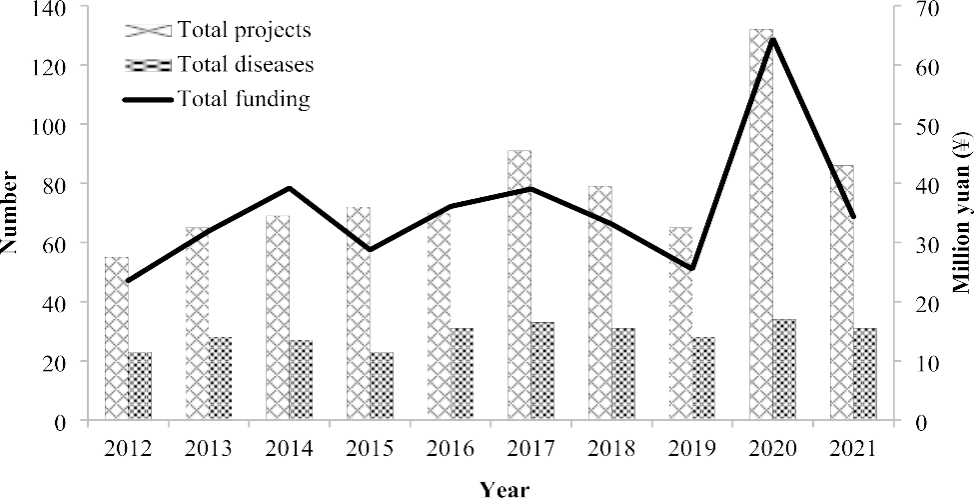
NFSC funding on rare disease and orphan drug R&D during 2012–2021

In China, funds for scientific research from the government can be granted at national (such as NSFC, National Scientific and Technology Support Fund, and the Fund for Key Projects of the Ministry of Science and Technology), provincial (such as the natural science funds granted by provincial science and technology departments) and ministerial (such as the natural science funds of the Ministry of Education) level [34]. The NSFC provides the greatest R&D input for rare disease research and orphan drug innovation [35]. In 2016, the guidelines from NSFC Medical Science Department encouraged “basic research on pathogenesis, prevention and treatment of rare diseases”. In 2021, special funds were established to support researches on rare cancers. During 2012–2021, a total of 784 projects covering 78 of the 121 rare diseases in the NLRD were funded, with cumulative total funding of ¥356.96 million ($53.36 million). There is a steady increase in annual funding and annual projects, with the year 2020 witnessing peaks in both of them (Fig. 1). The research topics range from pathogenesis to diagnosis and from drug screening to gene therapy and cell therapy. The top ten most-funded diseases are listed in Table 2, which are relatively less rare than other diseases in the NLRD. Among them, idiopathic pulmonary fibrosis, multiple sclerosis and generalized myasthenia gravis together account for about 30% of the total funding.

**Table 2:** Top 10 most-funded rare diseases by NFSC

No.	Rare disease	Total Funding (million yuan)	Total projects	Funding per project (million yuan)	Percentage (%)
1	Idiopathic Pulmonary fibrosis	40.30	95	4.24	11.32
2	Multiple Sclerosis	36.22	93	3.89	10.17
3	Generalized Myasthenia Gravis	34.77	73	4.76	9.77
4	Retinitis Pigmentosa	28.86	55	5.25	8.11
5	Amyotrophic Lateral Sclerosis	28.63	69	4.15	8.04
6	Hemophilia	16.45	43	3.83	4.62
7	Homocysteinemia	15.15	19	7.97	4.26
8	Neuromyelitis Optica	14.50	33	4.39	4.07
9	Progressive Muscular Dystrophy	9.57	16	5.98	2.69
10	Retinoblastoma	9.49	24	3.95	2.67

Another major funding for orphan drug R&D in China is the National Major Scientific and Technological Special Project for “Significant New Drugs Development” from the Ministry of Science and Technology, which was implemented from 2008 to 2020 and aimed to enhance China’s capability of independently innovating significant drugs. During that period, ¥ 68.23 million ($10.2 million) has been invested in a total of 14 projects of rare diseases, including hemophilia, myasthenia gravis, multiple sclerosis, idiopathic pulmonary fibrosis, amyotrophic lateral sclerosis, etc. It is encouraging to see that some progress has been resulted from the funded projects. For instance, Pirfenidone Capsule for idiopathic pulmonary fibrosis as well as Human Prothrombin Complex Injection and Human Coagulation Factor VIII Injection (freeze-dried) for hemophilia have been granted new drug certificate, filling the gap in domestic market.

### The implications on Orphan Drug approval in China

**Fig. 2:**
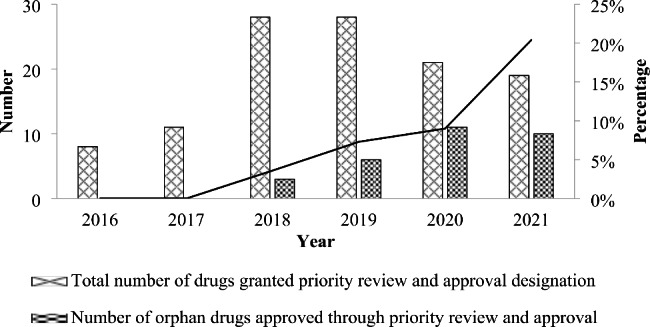
Priority review and approval of orphan drugs in China during 2016–2021

Due to the severe backlog and delays in drug application and approval before 2015, the effect of incentive policies targeting orphan drug approval process was very limited, resulting in very few approvals. It was not until 2016 when a range of fundamental reforms in drug regulatory framework were carried out consecutively that the approval of orphan drugs has been accelerated. Since 2016, totally 96 orphan drugs have been granted priority review and approval designation in China. Although no orphan drug with this designation was approved in 2016 and 2017, the percentage of approved orphan drugs in the total number of drugs with the same designation increased from 3.6% in 2018 to 20.4% in 2021 (Fig. 2). By the end of 2021, a total of 30 orphan drugs had been approved for marketing in China through this regulatory pathway.

**Fig. 3:**
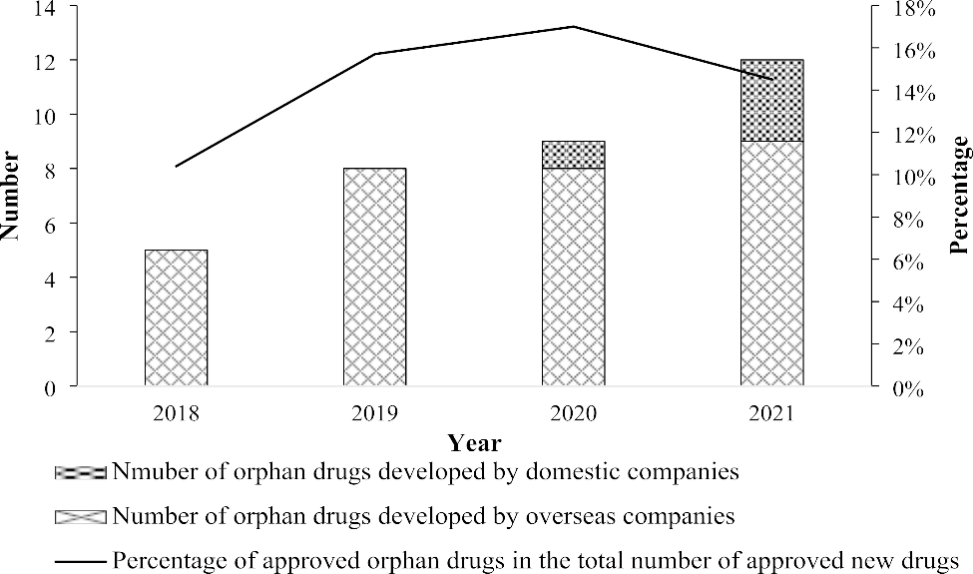
Orphan drug approval in China during 2018–2021

In addition, the total number of approved orphan drugs keeps growing steadily from 2018 to 2021, and the percentage of approved orphan drugs in the total number of approved new drugs of the same year has also dramatically increased, ranging from 10.4% in 2018 to 17.0% in 2020 in spite of a slight decrease in 2021(14.5%) (Fig. 3). Most of these drugs were developed by overseas companies, which is mainly attributed to the incentive policies specifically designed for imported drugs. Nevertheless, it is encouraging to see the attempts of some domestic pharmaceutical companies, illustrated by the approval of 1 and 3 domestic drugs in 2020 and 2021 respectively. It indicates that domestic drug innovators have begun to actively engage in orphan drug development through research collaborations with overseas big pharma and biotech companies.

### The implications on Orphan Drug Innovation

**Fig. 4:**
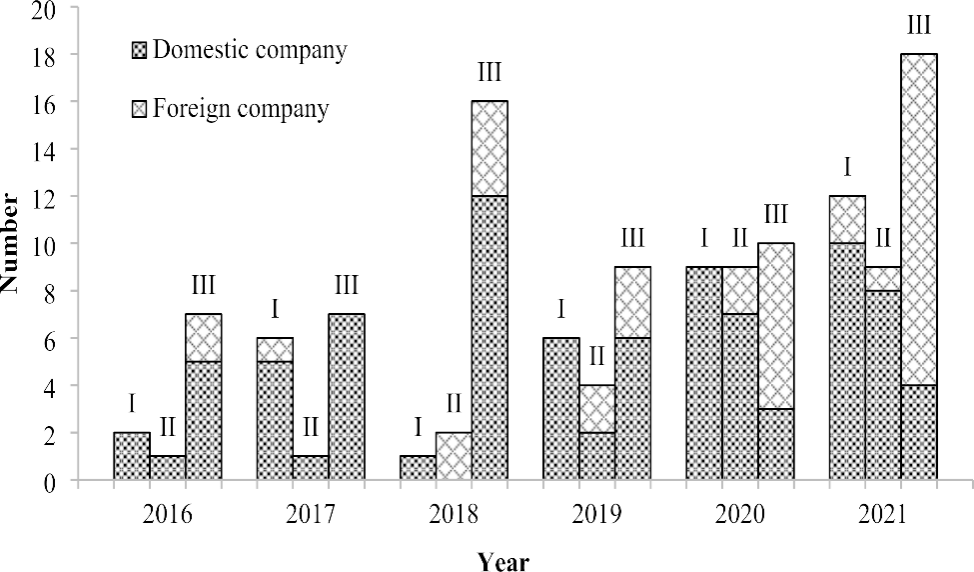
Approved clinical trials (phase I-III) of orphan drugs in China during 2016–2021

**Fig. 5:**
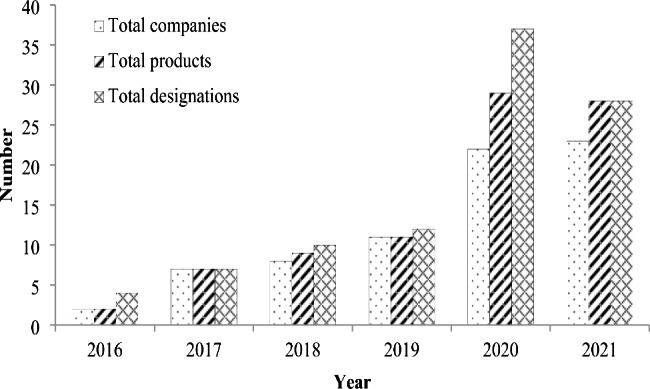
Orphan drug designation granted by FDA to Chinese pharmaceutical companies during 2016–2021

Driven by more concrete steps taken by NMPA to address the challenges in orphan drug development, an increasing number of domestic companies have been attracted to this field, which is reflected by the steady increase in the number of NMPA-approved orphan drug clinical trials conducted by Chinese pharmaceutical companies during 2016–2021 (Fig. 4). In this figure, it is also worth noting that there is a dramatic increase in phase III trials conducted by foreign companies, which are mainly multi-regional clinical trials (MRCTs) approved in China. MRCTs are more desirable for orphan drug development in terms of reducing the number of trials and subjects needed and accelerating drug access. Taken the data of domestic and foreign companies together, notable increases have been seen in all phases of clinical trials, and the types of rare diseases covered in those trials increased from 4 in 2016 to 13 in 2021. Some domestic companies even specialize in tackling rare diseases and begin to compete in the international market. During 2016–2022, the FDA granted a total of 98 orphan drug designations to 86 products developed by 73 Chinese pharmaceutical companies, with the year 2020 witnessing the largest number of total designations (37) and total products (29) and the year 2021 witnessing the largest number of total companies (23) (Fig. 5). These designations cover 47 indications, 32 (68%) of which are cancers. The top three companies with the most designations are Ascentage Pharma (13), BeiGene (9) and CSPC Pharmaceutical Group (4). With more domestic companies setting their sight on rare diseases and enhanced innovative capacity, China is expected to take more important roles in fulfilling the unmet medical needs for patients with rare diseases worldwide.

## Discussion

Huge investment, high failure rate and time-consuming researches are the common challenges faced by R&D of new drugs. Nevertheless, there are even more obstacles to the R&D of orphan drugs. The understanding of the pathophysiological mechanisms of rare diseases is always insufficient; the small patient population makes it difficult to recruit enough subjects to conduct clinical trials and obtain statistically powerful data; and the revenue from a niche market can barely recoup the development cost. Therefore, without incentives from government, profit-oriented pharmaceutical companies may not choose to enter this field. Since the 1980s, some developed countries/regions like the US, EU and Japan have established various policies to stimulate the development and accessibility of orphan drugs. Although China’s efforts in this aspect started late, policies aimed at promoting the R&D and industrialization of orphan drugs have been introduced at an ever-increasing rate in recent years, especially since 2012 when the *12th Five-Year Plan for National Drug Safety* was issued to encourage orphan drug R&D [14].

Before 2015, the availability of new drugs in China was compromised by the severe backlog of applications and delays in approval [36]. In 2015, more than 21,000 applications were on the waiting list for review, which was far beyond the capacity of the CDE staff [37]. The average delay for an applicant to register for a clinical trial of an innovative drug was 14 months (versus 30 days in the US), and the average time for review and approval was between 15 and 40 months (versus about 12 months in the US), leading to an average of 36-month lag between the US FDA and the former NMPA [38]. In order to prioritize orphan drug development, several policies were issued to accelerate the review and approval process. In particular, the *Announcement on Certain Relevant Policies on the Review and Approval of Drug Registration* issued in November 2015 set up a separate queue for orphan drugs [17]. However, due to the lack of fundamental reform in the regulatory framework, the effect of these policies on the approval process of orphan drugs was limited.

Starting from the *Opinions on Solving the Backlog of Drug registration Applications and Implementing Priority Review and Approval* issued in February 2016, a range of reforms were carried out to improve the efficiency of drug approval process [18]. Applicants of orphan drugs can apply for a clinical trial waiver or an agreed decrease in trial subject numbers, which significantly reduces the cost and restrictions of clinical development. Expedited approval pathways, especially priority review and approval, can be applied to orphan drugs to speed up the approval process and make them available as early as possible to fulfill the unmet medical needs. It is worth noting that some incentive policies before 2019 focus on the approval of orphan drugs marketed overseas, probably because most innovative drugs for rare diseases are initially developed abroad. These drugs can be exempted from racial difference trials and approved for marketing based on complete or partial acceptance of the clinical data obtained outside China. The NMPA may grant conditional approval while the sponsor completes commitment for clinical trial(s) based on NMPA’s review and conclusion.

One important highlight of those incentive policies is that China is moving towards robust data exclusivity for orphan drug development. Both patents and data protection provide competitive advantages for pharmaceutical inventions. Patent protection for medical products was first introduced in 1992 to implement the US-China Memorandum of Understanding signed in that year [39]. However, this was the only form of protection available for medical products in China until 2001. Since China’s entry into the WTO in 2002, innovative drugs have been entitled to six years of data protection. The *Opinions on Deepening the Reform on Review and Approval System and Encouraging Innovation on Drugs and Medical Devices* issued in 2017 allows applicants of orphan drugs to apply for the protection of independent and yet undisclosed study data, whereby marketing application of the same medical product developed by other applicants will not be approved during the data protection period [21]. A 10-year data protection for original orphan drugs was proposed. For further implementation, the NMPA issued the *Interim Measures for Implementing Pharmaceutical Study Data Protection* in 2018 [23], which clearly included orphan drugs in the scope of data protection. Although the proposed data protection period for original orphan drugs is decreased to six years, that for biologics is increased to 12 years. This is also an incentive to orphan drug developers since 80% of rare disorders are genetic in origin and biologics are most promising for the treatment [40]. In the 2022 draft of *Implementing Regulations of the Drug Administration Law of the People’s Republic of China*, a seven-year market exclusivity was proposed for approved orphan drugs, which is moving closer to the counterpart policies in developed countries [32]. However, further clarification of the scope of data exclusivity protection and the compatibility with newly enacted regulations is needed from NMPA for effective implementation.

Huge R&D spending is needed for the development of orphan drugs. Given that pharmaceutical industry is mainly responsible for the R&D of new drugs, the allocation of resources on rare diseases is much less than on non-rare diseases due to the much lower expected return on investments. In view of this, special funds from the government are necessary to foster the initiatives in orphan drug R&D. Compared with developed countries, the financial support on orphan drug development in China is still far from sufficient. In the U.S., funding from National Institute of Health (NIH) for rare disease research grows each year, reaching a total of $6.08 billion in 2021. In addition, the Office of Orphan Products Development (OOPD) has awarded more than 700 grants to conduct clinical trials of medicals products for rare diseases since the program’s inception in 1983 [41]. The amount of funding was $500,000 in 1983 [42]; however, it was followed by steady annual increase and reached more than 15, 16 and 25 million dollars in 2019 [43], 2020 [44] and 2021[45] respectively. Therefore, the input in orphan drug R&D from the Chinese government still needs to be further enhanced.

Driven by the above incentive regulatory policies and funding initiatives from the government, orphan drug development in China is growing rapidly with a steady annual increase in orphan drug approval and enhanced capability of orphan drug innovation. Nevertheless, the orphan drug industry in China is still in its infancy, we need to face up to the problems to be addressed for future success:

i. The lack of systematic legislation on rare diseases and orphan drugs.

Many developed countries such as the US, Japan, Australia and most EU member countries have legislative activities on rare diseases, which provide a series of incentive policies for the development, production, marketing, pricing, data protection, and funding of orphan drugs [46]. Taking the US as an example, its first major law on orphan drugs, the Orphan Drug Act, was enacted in 1983, offering pharmaceutical companies research funds, tax credits, fee waivers, and a seven-year market exclusivity to stimulate orphan drug development. The Rare Disease Act in 2002 established the Office of Rare Diseases at the NIH to stimulate and coordinate research in rare diseases. In addition, legislation for single rare disease entities has also been established, such as the MD-Care Act [47]. Although many incentive policies have been implemented in China, they are sporadic and fragmented and none of them has been elevated to the level of legislation. For this reason, the effectiveness of those reforms has not been brought into full play. China should draw on international experience to enact systematic legislation on rare diseases and orphan drugs to clarify the responsibilities of governments at all levels and relevant departments in the prevention and treatment of rare diseases, establish the definitions for rare diseases and orphan drugs and formulate procedures for their designation, stipulate the review procedure of drugs and medical devices for rare diseases, provide incentives for R&D and manufacturing of orphan drugs, improve medical insurance, medical assistance and other channels to ensure accessibility of orphan drugs and establish clinical pathway for diagnosis and treatment of rare diseases. By doing this, China can further promote the legalization and standardization of rare disease policies and better protect the rights and interests of rare disease patients.

ii. The lack of a dedicated orphan drug office.

In order to regulate and promote the development of orphan drugs and to centralize management resources, the US, EU, and Japan have all established specific orphan drug regulatory authorities. In the US, the OOPD of FDA and the Office of Rare Disease Research (ORDR) of NIH are responsible for the evaluation and approval of orphan drugs and the supervision and promotion of rare disease research respectively. The establishment of a dedicated orphan drug office at the national level will facilitate orphan drug designation, promote efficient and well-coordinated administration of orphan drugs from early R&D stage to marketing, and speed up the formulation of more targeted incentive policies. It is also suggested to establish an orphan drug review and evaluation committee led by the dedicated authorities and consisting of academic institutions, medical institutions, pharmaceutical companies and patient advocacy groups to participate in the review of orphan drugs and provide advice and consultations for clinical trials of orphan drugs in a more scientific and targeted manner.

iii. Insufficient financial support.

Apart from small market share, the requirement of more R&D input and more advanced scientific instrumentation further increases the investment risk on the part of orphan drug sponsors. Consequently, financial incentives from the government are always necessary. Despite the steady annual increase in NSFC funding in recent years, it only accounts for about 10% of similar funding in the US. In addition, the channel of financial support is mainly limited to research funding from the government. In contrast, financial subsidies for clinical and non-clinical research, tax credits, waivers of application fees, reduction in corporate tax and market exclusivity that are commonly exercised in western countries to reduce the R&D cost of orphan drugs are still lacking in China. For instance, tax credits can cover up to 50% of research and development costs in the US with up to $30 million per year in R&D grants provided for phase I through III clinical trials [48], while Japan offers a 15% tax credit for research expenses excluding financial subsidies and up to a 14% reduction in corporate tax [49]. China should also implement combined financial incentives to further encourage R&D initiatives.

## Conclusions

The low availability of orphan drugs has been a long-standing dilemma for rare disease patients in China, which calls for more urgent improvement compared with developed countries. With increased awareness of rare diseases among the public and stronger determination of the government, China has actively explored a variety of incentives to promote orphan drug R&D in recent years. While drawing on the experience of developed countries, the government has attempted to create a unique “Chinese model” to address rare diseases in line with its national condition and stage of national development. China has changed the status quo of orphan drug development by promulgating incentive regulatory policies for orphan drug innovation, investing more in basic and applied researches and mobilizing a wider range of social resources. It is worth noting that the number and types of orphan drugs approved for marketing in China are increasing year by year, more and more basic researches and clinical trials are being initiated, and more domestic pharmaceutical companies have announced their entry into the field of orphan drug innovation. In conclusion, China is working toward a more standardized and comprehensive rare disease ecosystem and is anticipated to play an increasingly important role in the global fight against rare diseases.

## Data Availability

The datasets analyzed in the current study are all available on the websites indicated in the subsection of “Data retrieval”.

## Abbreviations

NMPA: National Medical Products Administration
CDE: Center for Drug Evaluation
SAMR: State Administration for Market Regulation
FDA: U.S. Food and Drug Administration
NSFC: National Natural Science Foundation of China
NLRD: National List of Rare Diseases
MRCT: Multi-Regional Clinical Trial
NIH: National Institute of Health
OOPD: Office of Orphan Products Development
ORDR: Office of Rare Disease Research.
